# The systemic marginalisation of long-term casualised researchers in UK higher education

**DOI:** 10.3389/fsoc.2025.1626458

**Published:** 2025-10-08

**Authors:** Cecile B. Menard

**Affiliations:** ^1^School of Geosciences, University of Edinburgh, Edinburgh, United Kingdom; ^2^Institute for Academic Development, University of Edinburgh, Edinburgh, United Kingdom

**Keywords:** precarity, casualisation, long-term researchers, early career researchers, research culture, employment equity and policy

## Abstract

While casualisation of academic labour has garnered significant scholarly attention, much has focused on “early career researchers” (ECRs), an all-encompassing term that masks the long-term precarity many academics face. This study challenges that narrative by centering long-term researchers (LTRs)—defined as those in casualised research roles for 8 years or more—who are overlooked in policy and discourse. Drawing on a survey of LTRs (*n* = 179) in UK universities integrating qualitative and quantitative data, this study examines their career trajectories, academic contributions and barriers to progression. The study highlights systemic and structural mechanisms within universities and funding bodies that marginalise and invisibilise LTRs, such as exclusionary career frameworks, exploitative hierarchies and lack of mentoring, as well as the normalisation of precarity as an academic “rite of passage.” The findings expose a disconnect between the value LTRs bring—e.g. when securing grants, sustaining research continuity, teaching and supervising—and the lack of recognition or progression routes available to them. It shows how widespread bullying and discrimination at the intersection of ageism, gender discrimination and caring responsibilities—experienced by 40% of participants—combined with trajectorism and the illusion of meritocracy entrench inequities in HE. This study calls for actionable policy interventions, such as formal recognition of LTRs as a distinct category, greater transparency on the true extent of casualisation and career opportunities that prioritise intellectual contributions over arbitrary employment status. Such sector-wide structural reforms are imperative to dismantle the very systems that enable and profit from the exploitation of precarious academic labour and to put an end to long-term insecurity in HE.

## 1 Introduction

In the United Kingdom (UK), only 12% of early career researchers (ECRs) secure a permanent academic position, with the remaining 88% pursuing careers outside academia or in non-university research sectors (Royal Society, 2010). Such statistics are not isolated to the UK; policy documents and higher education (HE) sector reports from several countries have confirmed a global pattern of stagnant permanency and rising casualisation reflected in the growing number of precarious academics and the lengthening duration of insecure employment (Auriol et al., 2013; Brechelmacher et al., 2015; National Academies, 2014; Frølich, 2018; OECD, 2021; UCU, 2021; Koens, 2024).

The rise of the casualised and precarious academic workforce has spurred a growing body of literature focusing on their experiences. Much of this literature centres on ECRs, particularly their identity construction and precarity. Studies highlight how ECRs emulate the identities of permanent academics (Nästesjö, 2022) while navigating tensions between collegiality and competitive career ambitions (Angervall et al., 2017; Skea, 2021), oscillating between compliance and resistance (Bristow et al., 2017; Courtois et al., 2020) and balancing aspirations for traditional academic careers with the demands of an entrepreneurial academic model where academics are expected to market themselves as products (Skea, 2021; Smith, 2017; Mantai, 2019). These challenges are exacerbated by the lack of agency inherent in precarious employment (Courtois and O’Keefe, 2015), the fear of “unbecoming” an academic after each rejection (Archer, 2008; Bonnello and Wånggren, 2023), and the detrimental impact of precarity and job insecurity on mental health (Courtois and O’Keefe, 2015; Guthrie, 2017; Hollywood et al., 2019).

However, the term ECR is itself problematic. It is an all-encompassing term that is variously applied to doctoral students, postdoctoral researchers or anyone on a precarious contract in academia, while also carrying ageist undertones when interchangeably being used to mean “young” academic (see Archer, 2008; Mula et al., 2022; Royal Society, 2024). Critical race and gender scholars have long emphasised the importance of developing precise language to visibilise marginalised groups and the oppression they face (Bourdieu, 2002; Butler, 2006; Davis, 2023); against this backdrop, the sector-wide acceptance of the term “early career researcher” is a deliberate misnomer (Menard, 2022; Bonnello and Wånggren, 2023). It masks the long-term nature of precarious employment by implying a stage within a recognised career timeline that 88% of so-called ECRs will never access (Royal Society, 2010). Challenges to this narrative are further compounded by the lack of data on career destinations and duration in insecure employment, with the only published figure, which relies on snapshot data, coming from a 15-year old report (Royal Society, 2010). In recognition of this gap, recent University and College Union (UCU) policy was voted to mandate the collection of such information (UCU, 2025).

With one-third of the precarious workforce estimated to remain in insecure roles for more than 10 years (Mellors-Bourne and Metcalfe, 2017), the narrative that postdoctoral positions are “temporary and developmental” (Woolston, 2020) does not reflect reality. The implications of referring to all precarious staff as “ECRs” extend beyond semantics, carrying tangible consequences for both research and policy. For instance, when I submitted an earlier study on long-term casualised academics (LTCAs)—a group often conflated with ECRs, but distinct in having endured precarious employment for 8 years or more—the editor of the first journal I submitted the paper to rejected it, deeming it too narrow in scope and of limited international relevance. This response underscores how the pervasive use of the term “ECR” obscures the realities of long-term precarity, rendering the experiences of LTCAs invisible and marginalising their struggles within broader academic discourse. In this study, which became Menard and Shinton (2022, hereafter MS22), we focused on research-only LTCAs and the personal and professional circumstances that led to and sustained long-term precarious employment. We identified three categories of these long-term researchers (LTRs), each characterised by distinct aspirations and employment histories. The first category shared the same aspirations as ECRs, making the existing literature on ECRs relevant to them. The second category became LTRs due to unplanned events—some fortuitous, others unfortunate—that hindered their ability to conform to strict academic expectations (e.g., interdisciplinary researchers or part-time workers with caring responsibilities or disabilities). The third category, unlike ECRs, consciously chose to sustain research-only careers despite the almost inevitable precarity this entailed. Some themes also recurred in all categories: expectations to perform tasks outside formal duties, inadequate or absent managerial support, the perceived prestige—or lack thereof—of certain fields, exclusionary funding eligibility criteria, and instances of bullying and discrimination. Our findings challenged the outdated career frameworks that failed to accommodate the realities of contemporary academic demographics and employment practices.

MS22 contributed to an emerging body of literature that distinguishes between ECRs and LTCAs and visibilises the specific struggles associated with long-term precarity. Courtois and O’Keefe (2015) were among the first to dispel the myth that precarity was confined to “young” academics when they revealed that the average age of their respondents in a study on casualised academic labour was 39. Spina et al. (2022) explored the unique challenges faced by older precarious academics, examining how job insecurity is experienced differently across life stages. Cairns (2024) took another approach and examined their use as factotums within HE institutions, highlighting their exploitation within the system. Bonnello and Wånggren (2023), themselves long-term casualised academics, subtitled their book “Generation Precarity,” in reference to the massification of long-term precarity that they describe as the “limbo that never ends.” O’Keefe and Courtois (2024), on the other hand, reflected on the ethics of researching academic precarity when themselves in permanent employment. Meanwhile, Schaller (2021) approached the issue from a different disciplinary perspective, conducting a cost–benefit analysis of transitioning LTCAs or “staff scientists” from precarious to secure contracts. Crucially, these studies span multiple geographical contexts (Australia, Ireland, UK, United States, and Portugal), further demonstrating that long-term precarity in academia is not a localised issue, but rather a systemic consequence of neoliberal university models.

Building on these insights, it is evident that addressing the sector’s deep reliance on long-term precarious employment demands a concerted effort to raise awareness among key stakeholders. A follow-up study to MS22 was necessary to deepen our understanding of LTRs and inform evidence-based policy interventions. This paper presents such a study and was guided by two research questions to address gaps in the literature: 1/What defines LTRs as a distinct group and what are the implications for understanding academic labour structures? 2/Are the structures perpetuating long-term precarious employment in UK HE shaped by systemic factors or by institution-specific practices?

To address these questions, this study drew on themes identified in MS22 to design a survey integrating quantitative and qualitative data, which was distributed to LTRs in multiple HE institutions (HEIs). Although the quantitative data may not be statistically generalisable (because 1/ there are no data on the total number of LTRs in UK HE 2/the participants were self-selected) most studies on casualisation in HE are based on qualitative data only (Bonnello and Wånggren, 2023); this one presents a rare attempt at complementing the qualitative data with descriptive statistics on different aspects of long-term precarity. Here, we argue that the recurring themes and patterns across institutions provide compelling evidence to challenge the systemic structures that perpetuate the invisibilisation of LTRs and to drive policy reforms that address long-term precarity in academia.

## 2 Methods

The themes addressed in the survey were informed by the findings in MS22. The survey was organised as follows:

1. Relationship with traditional career structure. Participants were asked about the positions they had held, applied for, or chosen not to pursue throughout their careers.
2. Value to research group. This section explored the participants’ contributions within their research group.
3. Obstacles to career path. This section examined whether participants perceived their career trajectories as having been shaped by obstacles and the specific types of challenges they had faced.
4. Visibility and recognition. This section invited participants to reflect on how they had sustained a research career—even if not formally recognised—and to share their perspectives on measures for research staff being debated as part of broader discussions about research culture.

While each theme was broadly approached in a distinct section, some questions in separate sections intentionally overlapped in order to test response consistency throughout the survey. There were 56 questions in total, but no participant would have answered all of them because of conditional branching.

The landing page indicated that the survey would take up to 30 min. The participants were given the option to complete the survey over multiple sessions while maintaining their anonymity by requesting a link to complete it at a later time. This flexibility aimed to reduce self-selection bias by allowing participants with limited time or demanding schedules to complete the survey over multiple sittings. The survey was designed and conducted on the Jisc Online surveys v2 platform. The survey was designed and conducted on the Jisc Online surveys v2 platform and was live from 9 June to 15 July 2022. It was distributed via the Researchers14 network, whose members are academic developers from 22 universities collectively employing 65% of the research staff population in UK universities (Researchers14, 2017). Network members promoted the survey via their internal channels, often through mailing lists directed at their research staff, in exchange of gaining access to aggregated data from the quantitative questions specific to their institution. To facilitate this, the respondents were invited to indicate their institution, but this meant that, to protect respondents from being identifiable, no data about protected characteristics or specific disciplines were collected. As a consequence, in the absence of intersectional data on the survey participants and given the lack of quantitative data on LTRs more broadly, standard methods for assessing participation, non-participation or self-selection biases (such as comparing the survey demographics against sector-wide demographics; Elston, 2021) could not be implemented.

Thirty-five closed-ended or multiple-choice questions provided the quantitative data to calculate descriptive statistics, with cross-tabulation used to explore relationships and patterns within and across different themes. Eight of these included a “other” option that opened a free-text box. These eight free-text boxes and 20 open-ended questions served to collect the qualitative data in order to (1) allow participants to expand on their answers (2) empower them to shape their own narrative (3) provide space for new themes to emerge (Braun et al., 2020).

The qualitative data were analysed in Nvivo R1 by conducting a hybrid thematic analysis (TA). The four themed sections of the survey described above informed the themes of the inductive TA, but coding also accommodated deductive TA. During the first coding iteration, each statement in free-text responses was assigned a code, which was mapped under one of the original four themes. Code commonality across themes was then examined to identify opportunities for merging existing themes or creating new ones. Two additional coding iterations followed: the first one to cross check new codes within the revised themes, the second one to finalise all codes and themes and to ensure consistency across the dataset. The final themes constitute the three sub-sections in Section 3. Of the initial four themes, themes 1 and 3 were retained, theme 2 was re-classified as a sub-theme of theme 1. Theme 4, which appeared, which appeared across multiple responses throughout the survey, was dispersed across the other themes. A new theme, “Interventions for positive change,” emerged through the deductive TA and was added to the final list. Note that, following Morse (1991), hereafter the term “participant” is used when discussing qualitative answers and “respondent” when discussing quantitative responses.

During the coding stage, I avoided making assumptions about underlying meanings in responses that appeared vague, recognising that both such assumptions, as well as the responses themselves, may be influenced by an academic framework that typically overlooks LTRs in its dominant narrative due to their nonconformity with established academic career paths. It is also important to recognise that LTRs’ perspectives and their ways of expressing them may be shaped by their marginalised status and that my own perspective, as an LTR, may also be influenced by this. As such, this study is framed through a feminist standpoint approach, based on the understanding that knowledge is situated and that marginalised individuals are uniquely positioned to understand the experiences of others within the same group (Harding, 1993).

## 3 Findings

The successful target response was set to 120; this target was exceeded by 50%, with a total of 179 eligible responses. The participants were employed in 17 different universities: 94% (*n* = 168) were based in Russel Group institutions, a self-selected association of research-intensive universities, 2% (*n* = 3) in other universities and 4% (*n* = 8) preferred not to say. Fifty-six percent (*n* = 100) of participants had been LTR for 8 to 12 years, 13% (*n* = 23) for 13 to 16 years, 13% (*n* = 24) for 17 to 20 years and 18% (*n* = 32) for more than 20 years. Although no disciplinary information was collected, free-text answers suggested that the majority of participants were employed in STEMM (science, technology, engineering, mathematics, and medicine). This aligns with previous studies indicating that most staff in long-term precarious research-only contracts are in STEMM while precariously employed staff in social sciences, humanities and the arts are more often on teaching-only contracts (Marques et al., 2017; MS22; O’Connor et al., 2023).

### 3.1 Relationship with traditional career structure

The traditional academic career structure assumes a linear trajectory between being a PhD candidate and obtaining a permanent teaching and research academic position. In between is conventionally described as the postdoctoral stage, portrayed as a temporary period when individuals transition from student to independence. Postdoctoral positions sit within externally funded projects secured by a principal investigator (PI) or can be secured via a fellowship that allows the researcher to conduct their own research and in which a senior academic acting as mentor will have an advisory role only. This section of the survey examined where participants situated themselves within this traditional structure at different times in their career.

#### 3.1.1 Academic appointments

**Figure 1:**
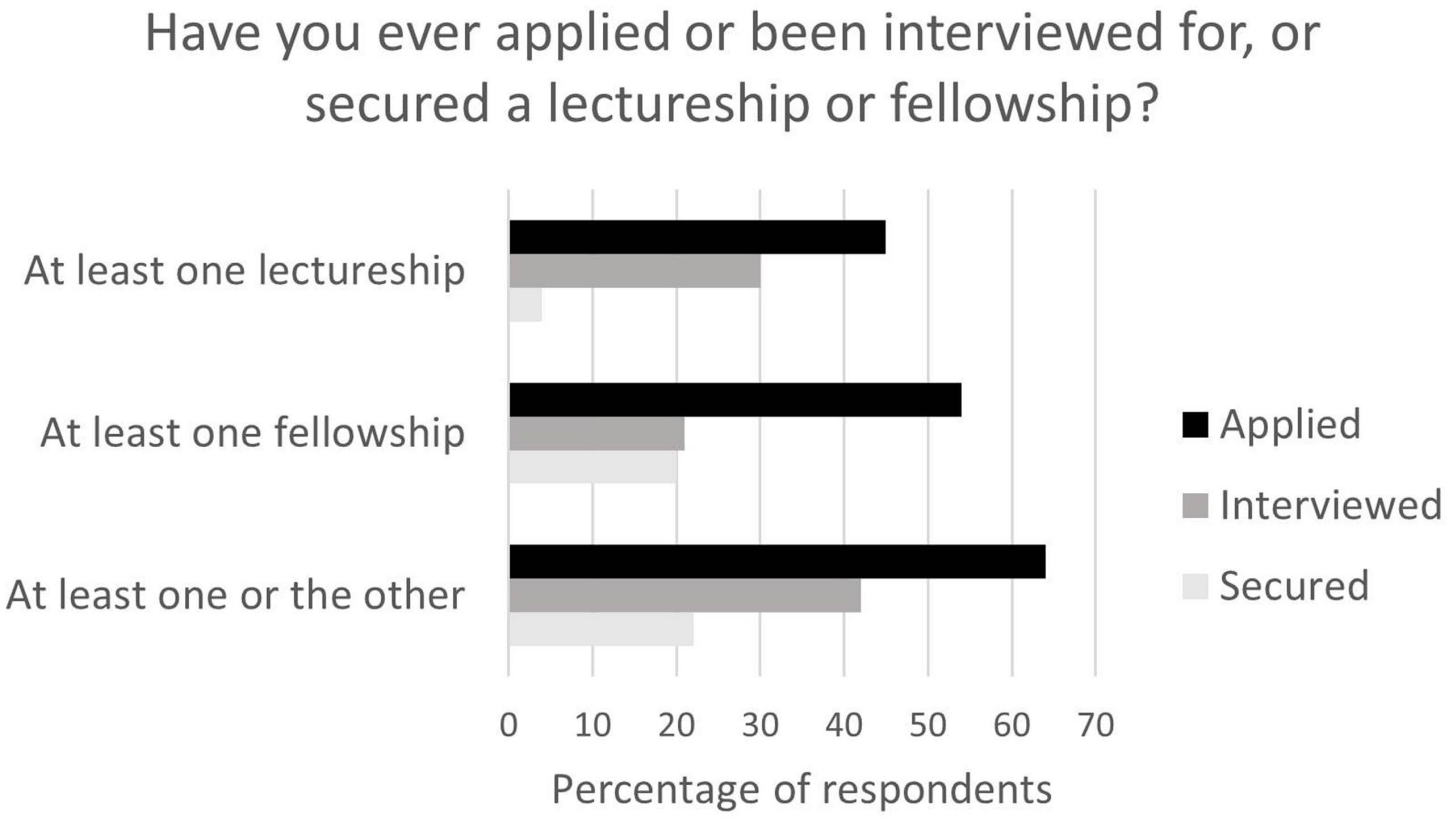
Percentage of respondents who applied, were interviewed for or secured at least one lectureship or fellowship.

The majority of respondents had applied for what is traditionally seen as the next stage in a postdoctoral researcher’s career, i.e., a fellowship or a lectureship (Figure 1), at some point in their career. The length of the working relationship with the PI did not significantly impact the respondents’ desire to have become independent. For example, of the 47 participants who had never worked with their current PI prior to their current project, 70% (*n* = 33) had applied for at least one fellowship or lectureship versus 62% of the 26 participants who had only ever worked with one PI. Of those who did apply, 16% (*n* = 15) for fellowship and 26% (*n* = 24) for lectureships had done so five times or more.

**Figure 2:**
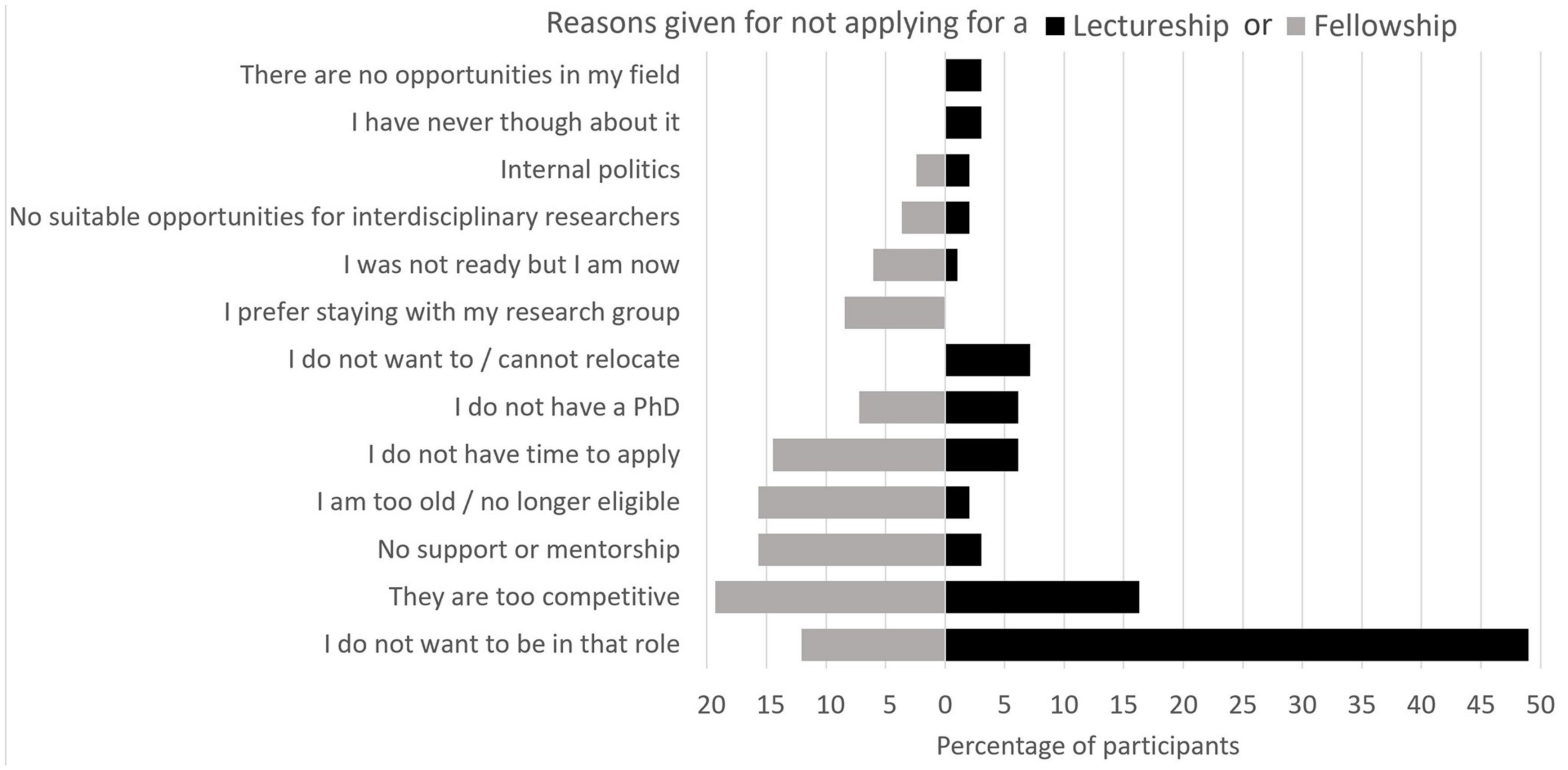
Reasons given by the participants for not applying for a lectureship or fellowship. The percentage shown is calculated as a proportion of those who did not apply, not of the total number of respondents.

Thirty-six percent of respondents had not applied for either. While some factors for not applying were consistent across both roles, others differed (Figure 2). Among participants who had not applied for a lectureship, the main reason was a clear disinclination towards that role (49%, *n* = 48). In contrast, the decision not to apply for fellowships was driven by a wider range of considerations; Table 1 provides exemplar quotes for reason cited by more than 10% of participants. These reasons were rarely isolated and intersecting circumstances often influenced the participants’ decisions not to apply for either. Personal circumstances, particularly caring responsibilities, played a significant role in many reasons cited in Figure 2 (i.e., “I do not want to be in that role,” “I do not want to relocate,” I do not have time to apply,” “I prefer staying with my research group”).

**Table 1:** Exemplar quotes of the reasons cited by more than 10 participants for not applying for lectureships or fellowships.

Reasons for not applying for lectureship or fellowship	Percentage, number of participants	Exemplar quote
I do not want to be in that role	*49%* *n = 48* *12%* (*n = 10*)	Lectureship: *“I have seen friends with lectureships who have no time for the research that they love, who spend long hours marking and involved in administrative jobs and whose research ends up squeezed. I do not want that.”* Fellowship*: “I like my current role as a researcher as part of a team”; “the idea of having to constantly apply for funding does not appeal to me”; “I dislike managing people”*
They are too competitive	*16%* (*n = 16*) *19%* (*n = 16*)	Lectureship: *“Never secured own funding, relatively lower number of publications compared to peers”; “working for long-term large scale projects with many members meant that my publication record never allowed this”; “I have no teaching experience and it’s extremely difficult to get any*.” Fellowship: *“Too little opportunities and too little time. The process is incredibly time consuming and with a tiny success rate”*
No support or mentorship	*19%* (*n = 16*)	Fellowship: *“I had no support from the department beyond ‘you should apply for this’—I had no idea what I was doing and I never saw an example.”*
No longer eligible	*16%* (*n = 13*)	Fellowship*: “Lack of mentorship at the beginning of my career meant I was unlikely to be successful. By the time I was, it was too late.”; “I changed my research scope after my PhD and by the time I had enough of a publication record to submit an independent fellowship application, I was at the end of the eligibility.”*
No time to apply	*14%* (*n = 12*)	Fellowship: “*Combination of regular work, family commitments (two small children) and finding the next job take up all my time and energy”; “my working time should be dedicated to what my pi says*.”

The percentage shown is calculated as a proportion of those who did not apply, not of the total number of respondents.

The survey also evaluated whether LTRs were involved in the core mission of universities: education. This is particularly important in the current academic landscape where few academic permanent positions are available: all else being equal, research staff with experience in teaching and supervision have a competitive advantage over those who do not (Nowell et al., 2020). Ninety-four percent (*n* = 168) of respondents had taught or supervised students while on a research-only contract. Of those, 41% (*n* = 68) did not obtain official recognition for their supervision work. The survey did not ask whether the participants had been remunerated for their contributions to teaching and learning; in retrospect, this represents a significant omission and limitation to understanding the value of some extra-contractual labour attributed to LTRS.

#### 3.1.2 Career expectations

**Figure 3:**
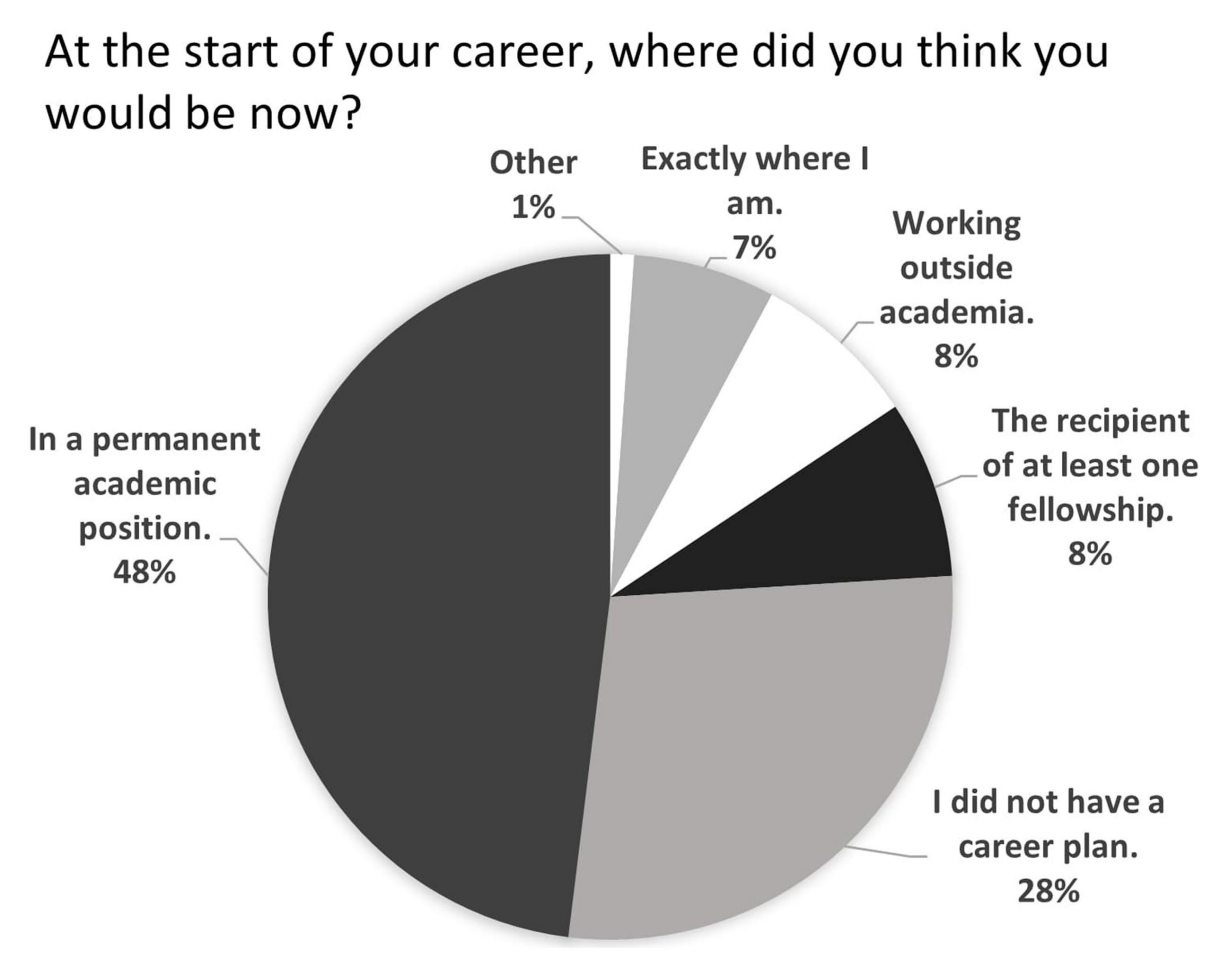
Distribution of the pre-defined answers to the question: at the start of your career, where did you think you would be now?.

Figures 3 and Table 2 illustrate the participants’ reflections on career achievements at two stages in the respondents’ career: now and at the start. When reflecting on what a successful career meant to them, the participants’ individual answers generally included multiple criteria, each synthesised between four categories in Table 2. The percentage of respondents who expected to have secured a fellowship (8%, *n* = 15) aligns with reported success rates: 8% in the US (National Academies, 2014) and 6% in the UK (Royal Society, 2010). More recent reports of higher success rates (e.g., 31% by UKRI, 2023) reflect a change in the application process; nowadays only the applicants pre-selected by their institutions following an internal competition can apply. In contrast, the percentage of respondents who had expected to have secured a lectureship considerably exceeds the percentage of academics who do: 48% (*n* = 86) of respondents, versus an estimated 12% (Royal Society, 2010). This disconnect may stem from the fact that all the permanent academics that researchers know have, by definition, secured permanent positions whereas the majority of research-only staff they know have not obtained a fellowship. This leads to a skewed perception of reality and a potential overestimation of the likelihood of achieving a permanent academic role.

**Table 2:** Exemplar quotes classified by categories identified by more than 10% of all participants in response to the question: “Thinking about your own professional goals, what would you define as being a successful career?”

Categories	Percentage, number of participants	Exemplar quote
Secure employment	*42%* (*n = 75*)	*“A job that would provide me with sufficient financial independence to get a mortgage and live comfortably.”* *“Enough job stability to set down roots and start a life outside of work.”* *“Not worrying about a contract ending and being given the formal position that everyone thinks I already have.”*
Good science	*31%* (*n = 55*)	“*Creating a new research field or at least changing the shape of an existing field”* *“It’s about contributing to tangible advancements in [my field], not about a job title or being the boss.”*
Be independent	*20%* (*n = 35*)	*“Working under own steam in the lab with ability to pursue own research ideas and publish work.”* *“Good publications and accomplished students and decent amount of research funding.”* *“Working on my own projects while supervising others, and where I would contribute to undergraduate teaching in some way.”*
Enjoy work	*17%* (*n = 30*)	*“A job you like, in an environment you like and that offers you a decent life-work balance.”* *“Success is happiness in the role”*

#### 3.1.3 Value to research group

The survey invited participants to reflect on their contributions and value to their PIs and research groups. One question used their contribution to the financial success of their PI or research group as a proxy to estimate their perceived financial value. A large majority of participants (86%, *n* = 153) had been named on a grant. Of those who had contributed to a grant proposal (83%, *n* = 149), over half (54%, *n* = 80) estimated that they had been the main or one of the main contributors to a grant, despite not being named as co-I or co-PI. Reasons for this varied, but the most frequently cited one was funding bodies requiring PIs to remain employed for a defined period beyond the project, therefore excluding those on project-based fixed-term employment.

Participants were then asked to reflect on the nature of their contribution to their research group or PI. Two distinct categories were identified although some participants cited both. Half of the participants (*n* = 90) estimated their value to lay in fostering cohesion, stability and maintaining group dynamics. They described a range of activities, which included the running of the lab, writing proposals, managing technicians or junior researchers, supervising MSc or PhD students, teaching, writing papers or engaging with industrial partners. Participants also emphasised their role as team players, collaborators, or providers of continuity within the group. Half of the participants (50%, *n* = 89) saw their value as being, instead, their individual expertise, often mentioning their “experience” and “specific skills.” The frequent mention of “experience” suggested that participants saw their value as being an LTR, i.e., bringing years of knowledge that an ECR would not have. Thirteen percent (*n* = 24) of participants cited their independence. This included those who noted they had secured their own funding (e.g., a fellowship), thus justifying their place in the broader research group (*n* = 9) and those whose experience allowed them to work with minimal or no supervision, allowing their PI to let them “get on with it” (*n* = 15).

### 3.2 Obstacles to career progression

**Figure 4:**
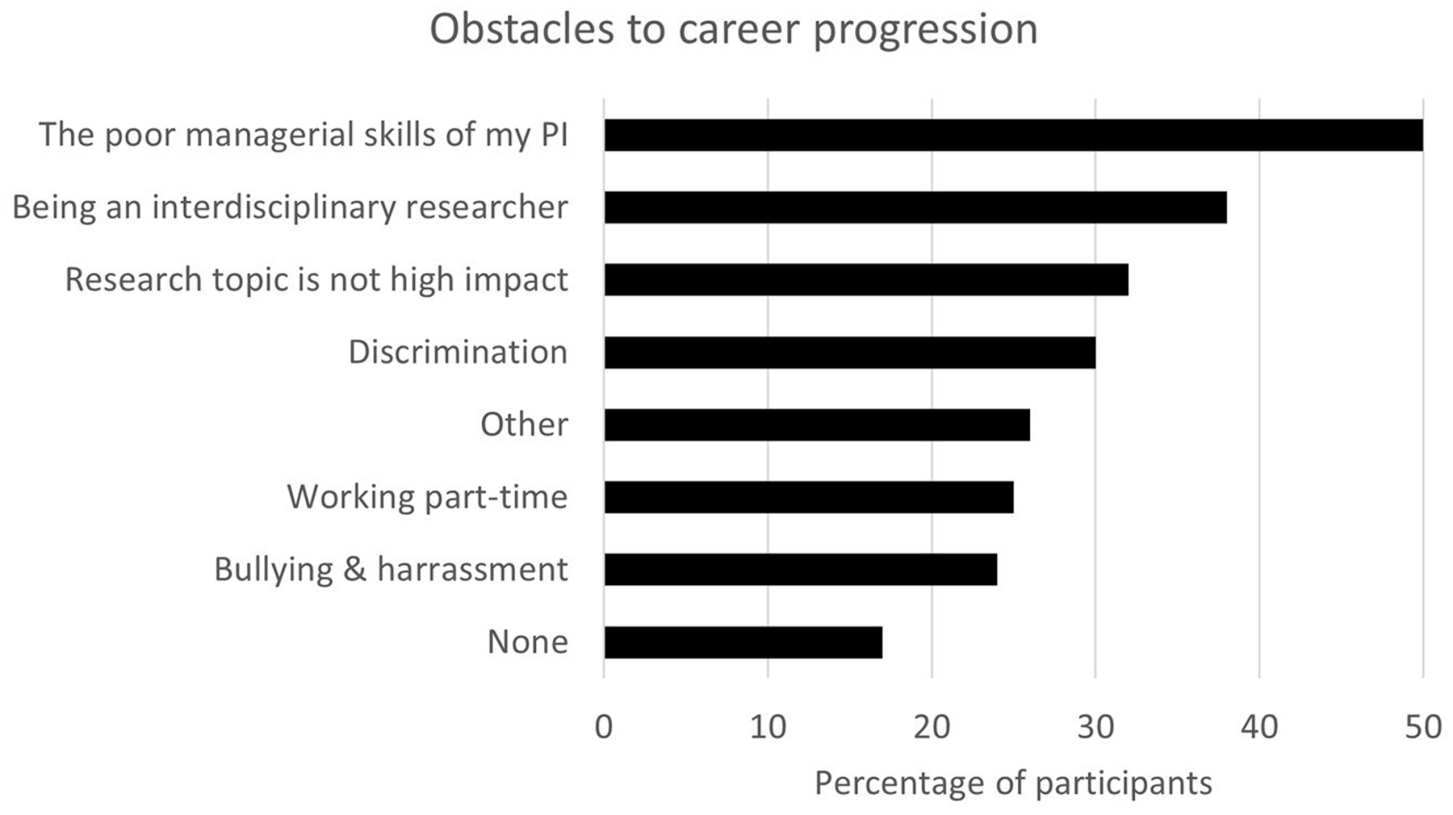
Obstacles to career progression reported by respondents. The answers were pre-defined based on previous findings and respondents could select multiple answers. As such, the total exceeds 100%.

The survey examined whether participants perceived their career trajectories as having been shaped by obstacles and the specific types of challenges they had faced. Respondents were presented with a list of potential obstacles informed by the findings in MS22 and listed in Figure 4. They were asked to select all relevant options and were also given the opportunity to provide additional information. The three most frequently reported obstacles are unique to the career structure and expectations within HE, suggesting that the HE sector operates under its own set of norms and systemic inequities. Other obstacles are not specific to HE, e.g., discrimination, bullying, harassment, and unfavourable treatment of part-time workers.

To provide some context, in the UK some characteristics (age, disability, gender reassignment, marriage and civil partnership, pregnancy and maternity, race, religion or belief, sex, sexual orientation) are protected by the UK Equality Act 2010, which makes it illegal to discriminate against individuals with these characteristics in the workplace and in wider society (Equality Act 2010, 2024). Equally, harassment linked to a protected characteristic is also unlawful under this act, but there is no law against bullying; its prevention falls under the remit of the employer (Gov.uk, n.d.).

To distinguish between general workplace issues and those specific to the HE sector, each is explored in a different subsection below.

#### 3.2.1 Discrimination, bullying and harassment

Nearly one third of participants (30%, *n* = 54) had experienced discrimination and nearly one quarter (24%, *n* = 42) bullying or harassment (B&H). Of the 42 who had experienced B&H, 26 also had been discriminated against, meaning that a total of 70 (40%) unique respondents had experienced discrimination, harassment or bullying. Of the 30% of respondents who had been discriminated against, half reported having faced intersectional discrimination. The predominance of age and gender as the characteristics most discriminated against (13%, *n* = 24, each) echoes other surveys investigating discrimination in academia and, more broadly, in the workplace (e.g., Ciphr, 2021; Emerald Publishing, 2022). Eighteen respondents (10%) had experienced racial discrimination, including towards their nationality. The number of respondents who were discriminated against because of disability, religion, sexual orientation or gender reassignment is low (4%, *n* = 8). However, as mentioned earlier, the survey did not collect information about participants’ protected characteristics so the significance of these numbers is difficult to assess because no information about the proportion of respondents who possessed these characteristics is available.

Respondents were also given the opportunity to list obstacles to their career that had not been listed in the predefined answers. One in five (20%, *n*= 35) cited having a family or caring responsibilities, which are not protected characteristics, i.e. twice as many participants who selected pregnancy or maternity (10%, *n* = 18), which are. Of particular importance is that the reasons given by the participants do not suggest that family and caring responsibilities prevent LTRs from doing their day-to-day activities, but rather that it is a normalised culture of working outside contracted hours, whether full or part-time, that prevents career progression. According to some participants, caring responsibilities “make it difficult for me to attend/present at conferences, which affects how my CV looks,” and “to juggle work hours, missing out on meetings [on non-work days means I am] seen as less important”; they “limit my ability to work outside my project duties to write grants and extra publications.”

When bullying happened, PIs were the main perpetrator although some respondents also cited co-workers and, more broadly, the toxic culture of the department they worked in, which allowed bullying to happen. When reporting the B&H, some respondents highlighted that they had not felt supported by Human Resources and, of the 21 respondents (12%) who did report B&H, only one experienced a satisfying resolution. Of particular importance, 42 out of the 43 who experience B&H either remained silent or continued working with the individual(s) who bullied or harassed them. The damage that B&H had on these respondents’ career, therefore, remains hidden and unseen.

#### 3.2.2 Academia-specific obstacles

Consistent with the findings of MS22, the poor managerial skills of PIs, whether current or previous, emerged as the most significant factor negatively impacting the participants’ careers (50%, *n* = 90; Figure 4). This issue manifested in two primary ways: poor people management and inadequate project management.

A substantial portion of participants (35%, *n* = 63) highlighted poor or unethical people management skills as a critical problem. Many reported that their PIs showed little interest in supporting their career development or lacked training in effectively managing researchers. Specific examples included PIs failing to assist with fellowship applications or grant writing, neglecting to acknowledge the informal contributions of LTRs—such as supervising PhD students or maintaining research group continuity—and imposing excessive workloads. While Section 3.1.3 notes that permanent academics often act as PIs on grants written by LTRs due to the latter’s ineligibility to apply, this dynamic can leave LTRs vulnerable to exploitation; some participants reported that their PIs claimed intellectual lead on grants despite minimal involvement. Additionally, PIs were described as either overly controlling—stifling researchers’ independence—or excessively hands-off—leaving LTRs without adequate guidance. Toxic work environments, unresolved conflicts and inadequate career advice were also cited, even when PIs were perceived as well-intentioned or generally supportive.

Poor project management was another significant concern (14%, *n* = 25), encapsulated by one participant’s observation: “There is a lack of ability to cut projects that clearly have significant obstacles to success.” Several participants found themselves involved in multiple projects with few tangible outcomes, as PIs either failed to see projects through to completion or lost interest midway, delegating responsibility to research staff without sufficient support. This lack of effective project management led to increased workloads and fewer publications, further hindering career progression.

The second most frequently listed obstacle in Figure 4 was being an interdisciplinary researcher (38%, *n* = 68). Participants’ experiences echoed those described in MS22, with many highlighting the difficulty of being perceived as “neither one nor the other.” Their specific skills, which they described as “seeing things differently” and synthesising broader concepts across multiple fields, was undervalued compared to mono-disciplinarity. They also reported facing significant challenges due to discipline-specific recruitment practices—“methodology unites my fields, [but] it’s hard to see on my CV”—or journal and grant reviewers—“Papers get rejected because reviewers from one discipline do not understand the objectives of the other.” Ironically, many LTRs become interdisciplinary by necessity for staying employed, driven by the sector’s reliance on insecure contracts. This creates a paradox: the very system that forces researchers to diversify their skills often fails to value those skills.

The third academia-specific obstacle was the participants’ research field not being suitable for publication in high impact journals. The distinction between having “a strong publication record in good journals [but] having nothing in top-tier journals” dramatically limited their career progression.

Finally, when given the opportunity to list obstacles that were not listed in Figure 4, 10 % of participants (*n* = 17) mentioned the rigidity of the academic career structure and the exclusionary eligibility criteria of many funding opportunities. For those who wanted to work for a PI or in a research group, “there [had] been no block to [their] career, other than there being no career path [for researchers] outside of becoming a lecturer.” For others, who aspired to become independent, they felt that HEIs and funders were “very poor at recognising and understanding atypical career paths—which tend to be very common in long-term researchers.”

### 3.3 Interventions for positive changes

While two questions in the last part of the survey specifically focused on interventions for positive changes, participants suggested interventions or changes that could positively impact their careers throughout.

#### 3.3.1 Participant perspectives

Three key interventions emerged from the answers (see Table 3 for exemplar quotes). One third of participants (*n* = 58) called for permanent or longer fixed-term contracts, citing professional benefits and highlighting the negative impact of precarity and insecurity on their mental health and on major life decisions. More secure contracts, they noted, would reduce time job seeking and applying for jobs, instead giving them space to delve more deeply in their current projects or enabling them to explore new directions in their field.

**Table 3:** Exemplar quotes of the reasons given to justify the interventions for positive change suggested by more than 10% of all participants.

Interventions for positive change	Exemplar quote
Permanent / more secure employment (32%, *n* = 58)	*“Taking the decision to have a baby whilst on a fixed-term contract felt incredibly risky and added a lot of stress.(…). [My precarity] has put strain on my relationship with my partners in times when we were waiting to hear if my contract was going to be extended or not. If felt so often like our lives were on hold.”* *“I really am on this anger/self-hate/system-hate kick at the moment. I think that I need help, but there is nowhere and no-one that I can turn to.”* *“The expectation that scientists should move around to gain skills is outdated. Moving to a new city or country is difficult, expensive, stressful and isolating.”* *“I spend quite some time searching out new opportunities and this can be quite stressful. If I had a permanent contract, instead, I could focus on the research itself”*
Recognition of LTRs within academic framework (26%, *n* = 46)	*“[there is a need for a] career progression framework that is more flexible and allows more varied career paths, that do not necessarily culminate in being PI.”* *“Researcher posts are only seen as valid when it’s a stepping stone to the next role so there’s a general assumption that if you are not following that model it’s because you are not good enough. As a result there’s not really much interest in making changes—after all it worked well for the people now in charge. So the first step has to be to challenge that assumption.”* *“I am aware of grant application reviewers querying the need to have ‘expensive’/experienced named researchers; I feel this reflects a lack of respect for the skills experienced researchers can provide.”*
Mentorship (10%, *n* = 18)	*“I had zero information about opportunities and did not know where to find the relevant info.” (migrant worker)* *“Because institutes rely so much on the PI-staff relationship, if this does not work, there is not anyone else official to talk to.”* *“Many of the things appreciated by fellowship review panels are extras to your day to day role. Awards, small grants, meeting attendance etc. If your PI is not a great mentor at encouraging you to apply, attend etc then these things tend to slip by. Particularly if you do not come from an academic background you may not know what exists or what is important without this help*.”

Frequently cited alongside the above intervention, was the need for recognition of the value of LTRs in university policies, particularly through the diversification of the academic career pathway (23%, *n* = 46). Half of these participants asked for official recognition of non-contractual activities (e.g., teaching), aiming to make currently invisible labour visible and to improve their career prospects. Other participants noted that recognition was necessary to tackle the culture of elitism in academia—including from funding bodies and grant reviewers—and to reduce stigma around LTRs. Measures currently considered by some HEIs and LTRs’ views on these are discussed in the next section.

The third intervention was reflexive: some participants felt their career path might have evolved differently had they received more support around career development at the start of their career. Mentorship independent of the PI, for reasons echoing the ones cited in Section 3.2.2., was the most common suggestion. At the start of their career, some participants had not understood how “to play the game” due to lack of mentorship; the implications for equity, diversity, and inclusion of this “insider culture” are particularly evident in responses from participants with protected characteristics (Table 3). The time required—and lost—trying to understand the unwritten rules of UK academic progression had been particularly damaging to their career.

#### 3.3.2 Institution-driven measures and policies

Respondents were asked about measures and policies that academic developers in the Researchers14 network reported were being discussed or implemented in their own institutions. The first measure, which is the most widely adopted among Russel Group universities (UCU, 2023), was the conversion of fixed-term contracts to open-ended contracts with review date (OERD). However, OERDs are fixed-term contracts in all but name: employment ends on the so-called “review date” unless additional project funding is secured (UCU, 2023). These strategically misnamed contracts only exist to satisfy government guidance, which requires that all fixed-term employees with more than 4 years employment be made “open-ended” (Gov.uk, n.d.). As such, although over half of respondents (53%, *n* = 95) were employed on OERDs, only one-third of all respondents, whether currently on a fixed-term or OERD contract, viewed this as a meaningful step towards more secure employment.

The second measure was the creation of a researcher pool where all researchers would hold permanent contracts and work where needed most, making the pool more about transferable skills than subject-specific expertise. This was the most popular measure (66%, *n* = 118) because it was the only one to guarantee permanency. However, the main objection to this model was summarised by one participant: “a ‘pool’ of researchers denies the fact that just as lecturers, we are specialised.”

The third measure was the creation of a research-only promotion pathway. Sixty percent of respondents expressed interest, but while half welcomed the opportunity for promotion regardless of contract type, the other half was interested only if this came with a permanent contract.

**Figure 5:**
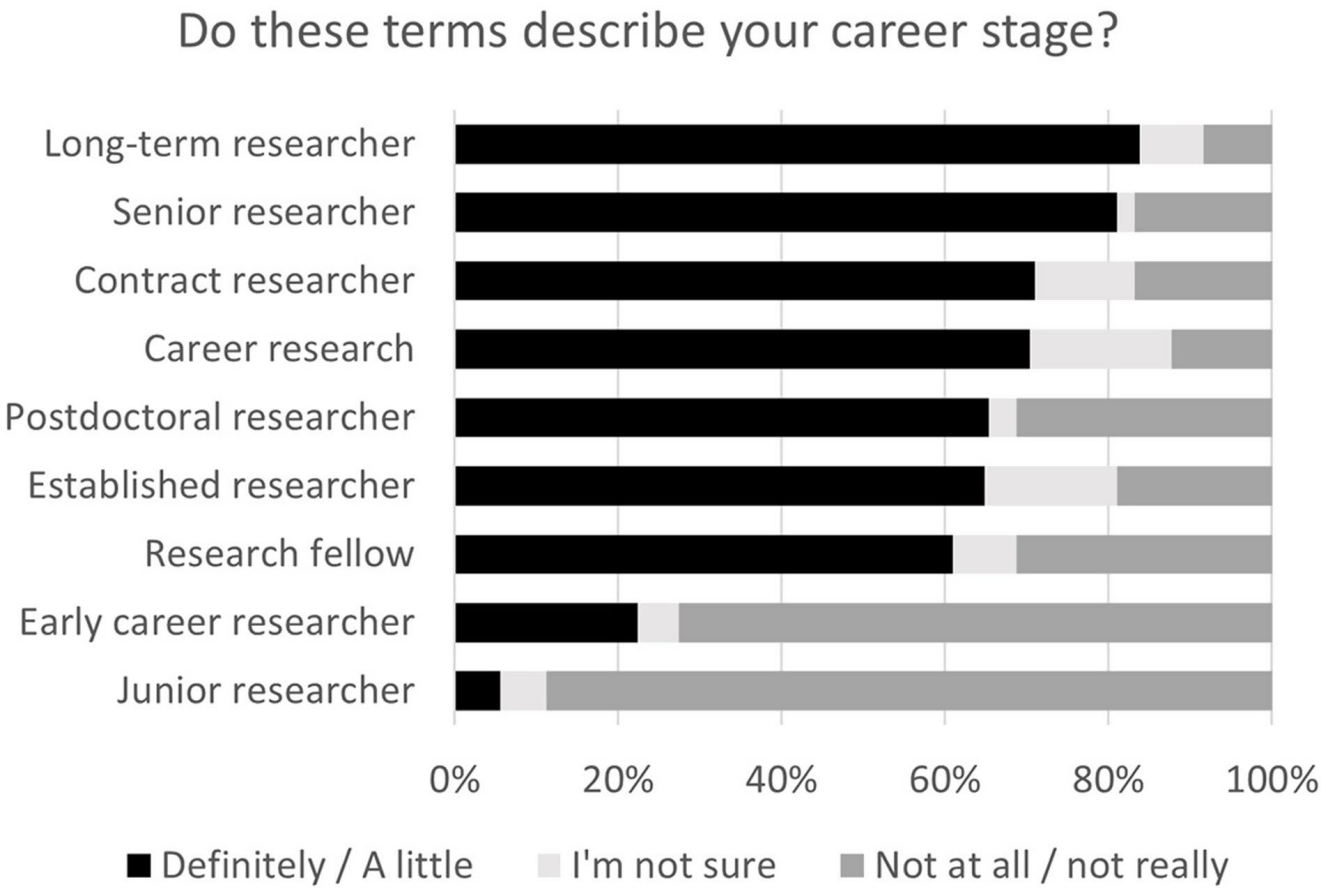
Respondents’ preference of terminology to describe their career stage. The answers were pre-defined and, for each term, each respondent had to tick one of the terms in the legend, i.e., “Definitely,” “A little,” “I’m not sure,” “Not really” or “Not at all”.

The fourth measure was the introduction of a new terminology to describe distinct groups among casualised and precarious academics. The participants were given a list of terms commonly used to describe their career stage and asked to tick all that they felt described their career stage (Figure 5). Although the term “long-term researcher” was first proposed by MS22 and is not widely used, it was most respondents’ preferred term. Of particular importance, more than two thirds of respondents did not relate to the term “early career researcher,” the one, arguably, most commonly used by universities and funding bodies. Also noteworthy is that three of the top four preferred options (i.e., long-term, senior and career researcher) convey a sense of recognition for seniority, experience and choice.

## 4 Discussion

This study is the first to obtain multi-institution quantitative and qualitative data on and to examine the issues faced by long-term researchers as a distinct workforce within academia. The stories of LTRs reveal structural and systemic mechanisms that facilitate their invisibilisation and marginalisation. Contrary to the dominant narrative, LTRs are not ECRs who stayed on too long: they are a workforce with distinct aspirations, contributions and challenges.

This study also challenges the academic discourse of postdoctoral and fixed-term research roles being transitional and necessary to develop an independent research agenda. The findings show that many LTRs already conduct independent research, but the structural barriers they face, such as exclusionary hierarchies and funding practices, undermine their professional autonomy and leave them open to exploitation. Other LTRs deliberately choose not to become independent. They play essential roles in maintaining the stability and functionality of research groups by managing day-to-day operations, supervising students and ensuring the continuity of projects, but rigid career structures deny them meaningful recognition or pathways for progression.

This study shows that the invisibility of LTRs is sustained through subtle forms of systemic violence. Academia replicates effective forms of oppression identified by feminist and critical race scholars (e.g., Lorde, 2017; Davis, 2023) that operate when marginalised groups internalise the values that equate legitimacy in a particular system. Marginalisation does not operate by overtly silencing the marginalised—this would paradoxically visibilise them—but by cultivating a narrative whereupon individuals unsanctioned by prevailing norms do not belong. Within our context, LTRs, who fall outside conventional academic career frameworks, are discouraged from asserting visibility, often self-marginalising because they themselves have internalised standards of merit and success.

Internalising this narrative is particularly damaging for identity construction because identity is constructed both actively and passively, i.e., by how individuals see themselves and how they are perceived by others (e.g., Välimaa, 1998; Clegg, 2008; McCune, 2019). When institutional cultures delegitimise roles that do not align with sanctioned career paths, those in such roles are compelled to construct their professional identities against dominant expectations. Academics often perform identities that align with institutional norms to secure recognition (Goffman, 1990; Archer, 2008; Nästesjö, 2022), but for many LTRs this is profoundly difficult because they occupy roles that are not formally recognised within academic hierarchies. They must reconcile a sense of professional legitimacy with institutional narratives that delegitimise their experience by misnaming them “early career” or that portray them as failed academics. The disconnect between how LTRs experience their value and how academia frames their worth undermines identity formation and contributes to broader professional disenfranchisement.

The pervasive ideology of meritocracy supports this structural marginalisation and obscures exclusionary practices. Institutions ostensibly reward talent and ambition, but, in reality, they define merit through narrow, normatively coded standards that reinforce gendered, racialised, ageist, and classed hierarchies (Castilla and Benard, 2010; Van den Brink and Benschop, 2014; Augoustinos et al., 2005; Martin, 2014; Jin and Ball, 2019). The illusion of meritocracy in the workplace is maintained because the mechanisms that feed non-meritocratic inequality are small incremental every day behaviours that are difficult to detect (e.g., van Dijk et al., 2020). Access to opportunities, such as training, enables the development of knowledge and skills, which leads to a cycle where those with greater access accumulate more rewards and recognition, thus amplifying existing inequalities. Our survey results align with these assertions and illustrate how LTRs face cumulative circumstances that deviate from the “typical” career trajectory, effectively excluding them from the meritocratic realm. Notably, poor mentorship was a recurring theme and was identified as the most significant obstacle faced by LTRs while discrimination based on age and gender was reported at equal rates.

The systemic privileging of linear trajectories is further reinforced by sector-wide policies. The UK Research and Innovation (UKRI), the public body of the UK government that directs research and innovation funding, values researchers transitioning towards “independence” and “on an upward trajectory” (UKRI, 2025). This framing not only dismisses contributions to team success, but, as Hammarfelt et al. (2020) note, defines merit as being simultaneously directional and velocity-based. This is problematic because while some career interruptions, such as parental leave, are institutionally recognised, other career-determining obstacles remain invisible. Notably, this study shows that a significant number of LTRs experienced bullying and discrimination. These obstacles are particularly consequential because the pervasive culture of impunity for perpetrators and enforced silence for victims, well documented by Ahmed (2021), further suppresses the possibility of redress and visibility. In such a system, speaking out is penalised and silence becomes a survival strategy, exacerbating inequality in a workforce where women and racially minoritised academics are already disproportionately employed on casual contracts (UCU, 2021). Although one key limitation of this study is the absence of equalities data, which constrains the analysis of some intersectional systemic inequalities (e.g., related to race or disability), the fact that patterns of disadvantage still emerged for individuals with intersecting identities shaped by age, gender, and caring responsibilities suggests the scale of these inequities is particularly pronounced.

This study shows that structural obstacles, systemic bias and institutional neglect shape career outcomes, but that, instead, individuals are held responsible for their own stagnation. The dominant ECR narrative—casting precarious academics as young, mobile and on the rise—erases those whose realities do not align with this template and further marginalises those already pushed to the periphery.

## 5 Conclusion

Although this study is grounded in the UK context, the findings have international relevance for higher education systems that share some structural mechanisms such as competitive funding environments, performance-based research assessments or high levels of casualisation (e.g., Australia, United States, Canada or Ireland). In these contexts, the mechanisms of invisibilisation, structural marginalisation and constrained career progression described here are likely to resonate with researchers beyond the UK (see O’Connor et al., 2023).

The findings in this study not only provide an important contribution to understanding the precarious workforce in academia, but they also have policy implications:

1. The HE sector should acknowledge the distinction between ECRs and LTRs and recognise LTRs as a separate category of researchers. Institutions and policymakers must reject the reductive categorisation of all research staff as ECRs and stop perpetuating misleading narrative about academic career trajectories.
2. HEIs and funders should work together—rather than deflect accountability to one another—to ensure that promotion, career opportunities and research proposals are assessed and/or funded based on merit regardless of contract type or years since PhD. Ownership and project leadership should be determined by intellectual contribution not employment status.
3. A structured support system, independent of principal investigators, should be established to provide mentorship and ensure that recognition is not contingent on a single individual. HEIs must also implement alternative promotion frameworks for research-only roles. These pathways should decouple career progression from the expectation of transitioning into teaching or administrative positions, recognising that many LTRs already contribute to the sector in non-traditional ways.
4. Policies recognising LTRs as a distinct demographic are essential to collect accurate equalities data and to deepen our understanding of how the sector’s workload crisis specifically impacts LTRs. Workload definitions must account for the unpaid labour expected of LTRs, which research culture deems necessary to remain competitive or to “prove” oneself worthy of the next contract. These expectations disproportionately disadvantage those unable to work beyond their contracted hours, particularly individuals with additional demands on their time, such as those with caring responsibilities-often women-or disabled researchers. Data collection must also push for greater transparency in the HE sector regarding the true extent of casualisation, beginning with challenging the legitimacy of open-ended contracts that function as fixed-term contracts (i.e., OERD). Such contracts enable universities to present themselves as better employers than they are, creating the illusion that they are addressing precarity and valuing staff loyalty. This misrepresentation of employment practices allows institutions to claim compliance with government policy—a claim that demands scrutiny.

The first of these policies is cost-neutral and should face little resistance from institutions, unless they are invested in continuing to obscure their employment practices, which, in view of this study showing that the marginalisation and invisibilisation of LTRs is deliberate, is the case. The second recommendation is also cost-neutral and could, in fact, benefit some institutions by broadening the pool of applicants eligible to secure funding for the institutions. However, its success depends on the implementation of the first and fourth policies, as well as a broader dismantling of the illusion of meritocracy. Without recognising how systemic inequalities shape career progression and access to opportunity, expanding eligibility alone will not be transformative.

Ultimately, all these policies would require a culture shift in HE. Ironically, after having contributed to creating what many see as a hostile research landscape (Gill, 2014; Arnold et al., 2016; Stern, 2016) the Research Excellence Framework (REF), the UK-wide assessment used to inform the allocation of around £2 billion per year of public funding for universities’ research (REF2029, 2025) is now calling for a change in the very culture it helped to create. Its newly introduced “People, Culture and Environment” component, which “offers a chance to recognise the environments that shape research excellence and ensure the UK’s system supports talent, innovation and impact” (REF2029, 2025), will account for 25% of the final score in the next REF cycle. In anticipation of this, funders and universities have been consulting academics and published strategies to improve their research culture for the past few years. However, a review of recent materials from the main UK funders, Royal Society (2024), UKRI (2023), and the Wellcome Trust (n.d.), reveals a striking omission: job insecurity and precarity are not addressed. This absence is difficult to justify, particularly when “unstable contracts and careers” emerged as the foremost concern in nine Wellcome Trust townhall meetings (Wellcome Trust, 2020). This disconnect suggests that while the policy recommendations proposed in this paper are designed to be realistic and achievable—even in a funding-constrained environment—a genuine culture shift remains unlikely within a sector that continues to serve institutional interests over the wellbeing of the people it employs.

At time of writing, 100 universities are going through redundancy and restructuring programmes (QMUCU, 2025) and hidden redundancies of staff on precarious contracts have been the first to happen (e.g., fixed-term contracts not being renewed or cuts in the number hours allocated to staff on hourly contracts. See Menard and Wånggren, 2025; Lai, 2025). In this context, even pragmatic recommendations are likely to be ignored in a sector that uses casualisation as a strategy to manage risk; precariously employed staff serve as a buffer against compulsory redundancies and budget cuts, rendered disposable in the name of institutional flexibility and risk aversion.

We must continue to challenge the structures and cultures that normalise casualisation, exploitation and inequity. The persistent inaction of decision-makers in HE, who have chosen to normalise and benefit from this system, demands that they are presented with concrete, actionable solutions—solutions that, if ignored, would expose their unwillingness to treat their employees fairly. The recommendations outlined here aim to bypass this inertia by proposing tangible steps towards meaningful change. However, the ultimate recommendation remains for the sector to reduce dramatically its reliance on precarious employment. This is not an aspirational goal, but an achievable reality—as demonstrated by so many other sectors that do not rely on precarious labour—that deliberate management choices over the past decades has denied. In view of the well-established causal link between the marginalisation of precariously employed workers and mental health issues (Irvine and Rose, 2022), the extent of casualisation in the HE sector contravenes legal and ethical obligations regarding workplace wellbeing.

Universities should serve as a public good, but for their workforce, they instead reflect the inequalities of wider society. Higher education policies must urgently evolve to reflect the realities of today’s research workforce by updating career structures that no longer serve the diversity roles within academia. Decision-makers, in particular, must take responsibility for addressing these systemic issues, as their choices perpetuate a broken system that undermines the very foundation of academic research.

## Data Availability

The datasets presented in this article are not readily available because they contain personal and potentially identifiable data. Requests to access the datasets should be directed to cmenard@ed.ac.uk.
